# Time Course of Cell Sheet Adhesion to Porcine Heart Tissue after Transplantation

**DOI:** 10.1371/journal.pone.0137494

**Published:** 2015-10-07

**Authors:** Dehua Chang, Tatsuya Shimizu, Yuji Haraguchi, Shuai Gao, Katsuhisa Sakaguchi, Mitsuo Umezu, Masayuki Yamato, Zhongmin Liu, Teruo Okano

**Affiliations:** 1 Institute of Advanced Biomedical Engineering and Science, Tokyo Women’s Medical University, TWIns, 8–1 Kawada-cho, Shinjuku-ku, Tokyo, 162–8666, Japan; 2 Translational Medical Center for Stem Cell Therapy, Shanghai East Hospital, School of Medicine, Tongji University, Shanghai, 200120, China; 3 Research Institute for Science and Engineering, Waseda, University, TWIns, 2–2 Wakamatsu-cho, Shinjuku-ku, Tokyo, 162–8480, Japan; The University of Adelaide, AUSTRALIA

## Abstract

Multilayered cell sheets have been produced from bone marrow-derived mesenchymal stem cells (MSCs) for investigating their adhesion properties onto native porcine heart tissue. Once MSCs reached confluence after a 7-day culture on a temperature-responsive culture dish, a MSCs monolayer spontaneously detached itself from the dish, when the culture temperature was reduced from 37 to 20°C. The basal extracellular matrix (ECM) proteins of the single cell sheet are preserved, because this technique requires no proteolytic enzymes for harvesting cell sheet, which become a basic building block for assembling a multilayer cell sheet. The thickness of multilayered cell sheets made from three MSC sheets was found to be approximately 60 μm. For investigating the adhesion properties of the basal and apical sides, the multilayered cell sheets were transplanted onto the surface of the heart’s left ventricle. Multilayered cell sheets were histological investigated at 15, 30, 45 and 60 minutes after transplantation by hematoxylin eosin (HE) and azan dyes to determine required time for the adhesion of the multilayered sheets following cell-sheet transplantation. The results showed that only the basal side of multilayered cell sheets significantly enhanced the sheets adhesion onto the surface of heart 30 minutes after transplantation. This study concluded that (1) cell sheets had to be transplanted with its basal side onto the surface of heart tissue and (2) at least 30 minutes were necessary for obtaining the histological adhesion of the sheets to the heart tissue. This study provided clinical evidence and parameters for the successful application of MSC sheets to the myocardium and allowed cell sheet technology to be adapted clinical cell-therapy for myocardial diseases.

## Introduction

In both congenital and acquired heart diseases, a large number of myocardial cells are damaged, and their normal functions are lost. Donated tissues and organs are often used for replacing damaged tissues and organs, but the need for transplantable tissues and organs is available supply. Recently, one of the clinical approaches to the treatment of heart disease is to repair and replace damaged tissues through injection of stem cells into the circulation or the direct transplantation of them into the injured heart tissue for improving cardiac functions and inducing the formation of new capillaries [1–3]. However, this approach has failed to demonstrate a significant potential in clinical trials. The stem cell populations that have been tested in these studies vary widely, because the injected cells are quickly washed away by blood flow and unable to easily integrate with the host heart tissue following transplantation [4, 5]. Moreover, the direct injection of the cells into the heart tissue is hazardous due to the possible blockages of the circulatory pathways, resulting in more life-threatening complications such as ventricular arrhythmia [6]. To overcome these problems, a new strategy for cell-based therapies is necessary. Therefore, it is important to improve the adhesion between transplanted cells and heart tissue. For enhancing adhesion, an innovative cell-sheet engineering technique with a temperature-responsive culture dish has been developed by the authors [7, 8]. Over ten years, cell sheets prepared by this technique have been applied to repairing various tissues and organs in animal studies as well as clinical trials including cornea [9, 10], heart [11–14], esophagus [15, 16], periodontium [17, 18], liver [19, 20], kidney [21], ect.

When culture temperature is reduced from 37 to 20°C, a monolayer cell sheet spontaneously detaches itself from the surface of temperature-responsive culture dish without using any proteolytic enzymes such as trypsin for the harvest. This culture method allows cell-cell connections and interactions to be preserved and maintains the presence of cells secreting and organizing extracellular matrix (ECM) components including some collagens, laminins, fibronectin, vitronectin, elastin and specialized proteins [22, 23]. ECM provides structural support for cell adhesion and tissue organization. Therefore, cell sheets can be directly transplanted onto the various kinds of tissues and organs without using any mechanical device such as suturing. This method is superior to single cell suspension injection, and the preservation of ECM in cell sheet regulates cell behavior by affecting cell survival, shape, proliferation, migration, and differentiation [24]. ECM is very important for the early stage of tissue regeneration.

In recent years, MSCs as one of the most common cell a sources have been investigated for regenerating complicated tissue and internal organs composed of multiple cell types [1]. Preliminary research in animals indicates that mesenchymal stem cells transplanted into damaged heart tissue can have beneficial effects [2]. The authors’ results have reported that adipose tissue-derived MSCs cultured on a temperature-responsive dish are able to generate a cell sheet, which can be used for repairing the infarcted myocardium of rats [11]. Although it has been explored in mouse and rat models, the small animal experiments are insufficient to extrapolate its results to humans. It is necessary that this application is shown to be feasible in larger animal models such as pigs. These results will encourage the use of this technique for the clinical application for the treatment of human heart diseases. It is also important to determine the required time for transplanted cell sheets to adhere firmly onto damaged heart tissue during open-heart surgery.

In this study, porcine bone marrow-derived MSCs sheets were cultured on temperature-responsive dishes, and the cell sheets obtained were transplanted to the left ventricle of heart. The adhesion between the cell sheet and the heart tissue was examined histologically for determining minimum required time to achieve a successful adhesion. In addition, the transplantation was also performed with either the basal side or apical side of the cell sheet in contact with heart tissue to verify which ECM component of the cell sheet was necessary to achieve the maximum adhesion. This study is the first report providing clinical evidence and parameters for the successful transplantation of MSC sheets to the myocardium, and information obtained in this work is a critical step in the development of cell sheet technology as a potential cell-based therapy for cardiovascular diseases.

## Materials and Methods

Animal care complied with the "Guide for the Care and Use of Laboratory Animals" (National Institutes of Health publication No. 85–23, revised 1996). Experimental protocols were approved by the Ethics Review Committee for Animal Experimentation of Tokyo Women's Medical University.

### Isolation and Culture of Porcine MSCs from the Bone Marrow

Male Clawn mini pigs (weight: 12–20 kg) (Japan Farm, Kagoshima, Japan) were used in this study. All experiments were conducted in accordance with Tokyo Women’s Medical University guidelines for the care and use of experimental animals. Bone marrow (5–10 mL) was collected from the ilium of the animals under general anesthesia that was induced with ketamine hydrochloride (5 mg/kg), medetomidine hydrochloride (80 μg/kg), stadol (0.2 mg/kg), atropine sulface (50 μg/kg) and maintained with 1 L/min N_2_O, 2 L/min O_2_, and 2 L/min isoflurane. Bone marrow was extracted by inserting a 16-gauge needle into the shaft of the bone and flushing with 3 mL heparin salt solution. Histopaqe–1077 (15–20 mL) was then added to the collected bone marrow (approx. 5 mL) and centrifuged at 1710 x g for 10 minutes at 4°C. After being washed twice with phosphate-buffered saline (PBS) to remove erythrocytes, the cells were then collected and cultured in 20 mL human mesenchymal stem cell growth medium, which consisted of 50 mL MCGS (Mesenchymal Cell Growth Supplement) (Cell Culture Tested, Lonza, Walkersville, MD, USA), 10 mL L-glutamine, 0.5 mL penicillin-streptomycin on a 100-mm dish at 37°C with 5% CO_2_ for 7 days. A small number of spindle-shaped cells appeared in visible symmetric colonies after 3 days culture.

### RNA extraction and Real time PCR

Total RNA of single and triple-layered MSCs cell sheet were isolated from cell pellets using TRIzol reagent (Invitrogen, Life Technologies, USA), according to the manufacturer’s instructions. The concentration of RNA was determined by spectrophotometric measurement after removal of potentially contaminating genomic DNA. Isolated RNA was then used as a template for reverse transcription and the synthesis of cDNA. The primer sequences for the ECM and angiogenic gene, *COL1A1*, *COL1A2*, *COL2A1*, *Fibronectin(FN)*, *LMNA*, *bFGF*, *HGF*, *VEGF* are provided in Table 1. Real-time PCR was performed using a SYBR Green-based PCR Master Mix (Applied Biosystems, Life Technologies, USA) and ABI7500 Real-Time PCR System (Applied Biosystems, Life Technologies, USA). *Gapdh* was used as a control.

**Table 1:** Sequences of primers in Real Time PCR.

Genes	Accession number	Primer	sequence (5’–3’)
*Gapdh*	NM_001206359.1	Forward	TGCCCCCATGTTTGTGATG
		Reserve	TGTGGTCATGAGCCCTTCC
*COL1A1*	XM_005668927.1	Forward	AGACATCCCACCAGTCACCT
		Reserve	TCACGTCATCGCACAACACA
*COL1A2*	NM_001243655.1	Forward	GGCTCTGCTACACAAGGAGT
		Reserve	CCCTTTCTTGCAGTTGCCTC
*COL2A1*	XM_001925959.5	Forward	GAAGTTGGACCTCCCGTGTC
		Resreve	CTCTCCTTGCTCGCCTTTGG
*Fibronectin*	XM_003133643.2	Forward	TCCCAGAGAAGTGGTCCCTC
		Resreve	GAGAGCTTCTTGTCCTGTCCT
*LMNA*	XM_005663272.1	Forward	TCCTACCTCCTGGGCAACTC
		Resreve	CATGAGGTGAGGAGGAAGCG
*bFGF*	XM_005666885	Forward	GCCATTCTACGTGAGCTGGT
		Resreve	ACCCCTCTCTCTTCTGCTTG
*HGF*	XM_0031130222.3	Forward	TTTGCCTTCGAGCTATCGGG
		Resreve	ACTTTCCCCATTGCAGGTCA
*VEGF*	NM_214084.1	Forward	TGCTCTCTTGGGTGCATTGG
		Resreve	ACCACTTCGTGGGGTTTCTG

### Flow cytometry analysis

MSCs were incubated with CD29, CD90, CD146, CD45, CD31, CD71 (BD Biosciences, USA) and analyzed using LSR II (BD Biosciences, USA) [25–27].

### Culture of Multilayered MSCs Sheet

MSCs populations were increased through the repeated passages of the cells by trypsinization (3 to 4 passages). Non-adherent cells were then washed during 2–3 fresh medium changes. The remaining purified MSCs populations were further expanded in culture and were confirmed to express CD29, CD90, and CD146 markers according to a reported method [11]. These bone marrow-derived MSCs (6.0 х 10^5^ /mL) were then cultured in MSCs growth medium (5 mL) on temperature-responsive dishes (UpCell) (CellSeed, Tokyo, Japan) at 37°C for 6–7 days until confluent culture was observed. The confluent cultured MSCs were then incubated at 20°C. Within 20–30 minutes, MSCs detached themselves from the culture dish and floated up into the medium as a monolayer cell sheet. The detached cell sheet shrank from 60 mm in diameter to approximately 20 mm because of their cytoskeltal reorganization [16]. After the sheet detached itself from the culture medium, the media was removed and cell sheets were re-incubated with 100μl culture medium for keeping wet at 37C for 30–40 minutes. The next cell sheet from another dish was then placed on the top of the first cell sheet allowing the basal side of the transported sheet to attach to the apical side of the first sheet, and the layered sheet was incubated at 37°C for other 30–40 minutes again. This process was repeated to add a third layer successfully preparing multilayered cell sheets.

### Transplantation of the Multilayered MSCs Sheet to Porcine Heart Tissue

The heart of the pig was exposed by midline thoracotomy under general anesthesia. The autologous porcine multilayered cell sheet was immediately collected by a sterilized polyethylene terephthalate film (3 × 4 cm, 3M A4 overhead projector (OHP) transparency film CG 3300) for *in vivo* transplantation. After open the chest and the pericardium, the autologous porcine multilayered MSCs cell sheet was directly transplanted onto the left ventricle of the beating heart with the basal side for 15, 30, 45 or 60 minutes. Saline (5–10 mL) was dripped onto the cell sheet after the transplantation to ensure that the cell sheets remained physically attached. Then the pericardium and the chest were closed in order. Using the same procedure, another cell sheet was also transplanted onto the left ventricular surface with the apical side attached to the surface of the heart tissue. In order to make the result more solid, five animals were used for each time-point for basal and apical side of cell sheet adhesion in our studies.

### Histological and Scanning Electron Microscope (SEM) Analyses

After the cell-sheet transplantation protocol as described above was completed, the cell sheets together with the underlying heart tissue were removed and fixed with 10% formalin for histological analysis. The tissue specimen including cell sheet was also fixed in 2% glutaraldehyde and coated with a nanometer thickness of conductive material on the surface (Neo super fine osmium coater, Meiwa, Japan), and then the specimens were observed by an electron microscope (S–4300 Scanning Electron Microscope) (Hitachi Hightechnology, Tokyo). Specimens were then embedded in paraffin and sliced into 5-μm-thick sections, which were treated according to a conventional method for hematoxylin eosin (HE) staining. The sections were also subjected to azan staining for discriminating the collagen-rich epicardium between the transplanted cell sheets and the heart tissue. All stained sections were observed by a conventional microscope (ECLIPSE E800) (Nikon, Tokyo Japan).

## Results

### Preparation and Characterization of Multilayered Cell Sheets

**Fig 1:**
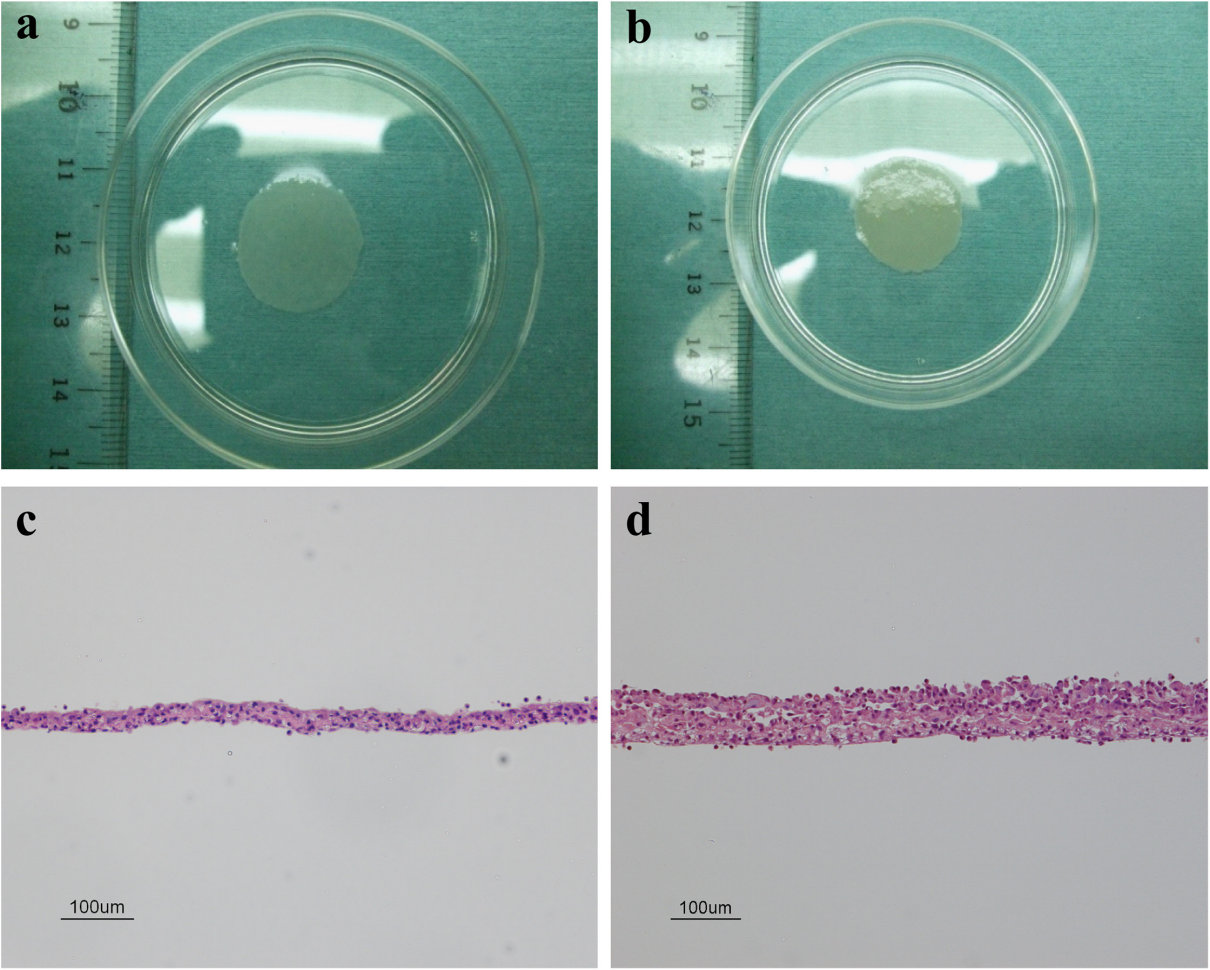
Photographs and the histological cross section of cell sheets from bone marrow-derived mesenchymal stem cells (MSCs). Top: (a) Cell sheet composed of a monolayer of MSCs, and (b) cell sheet composed of triple layers of MSCs. Each cell sheet was approximately 20 mm in diameter. Bottom: (c) The thickness is approximately 20 μm for a monolayer layer of MSCs, and (d) the thickness is approximately 60 μm for a triple layers of MSCs. Scale bars in c–d are 100 μm.

**Fig 2:**
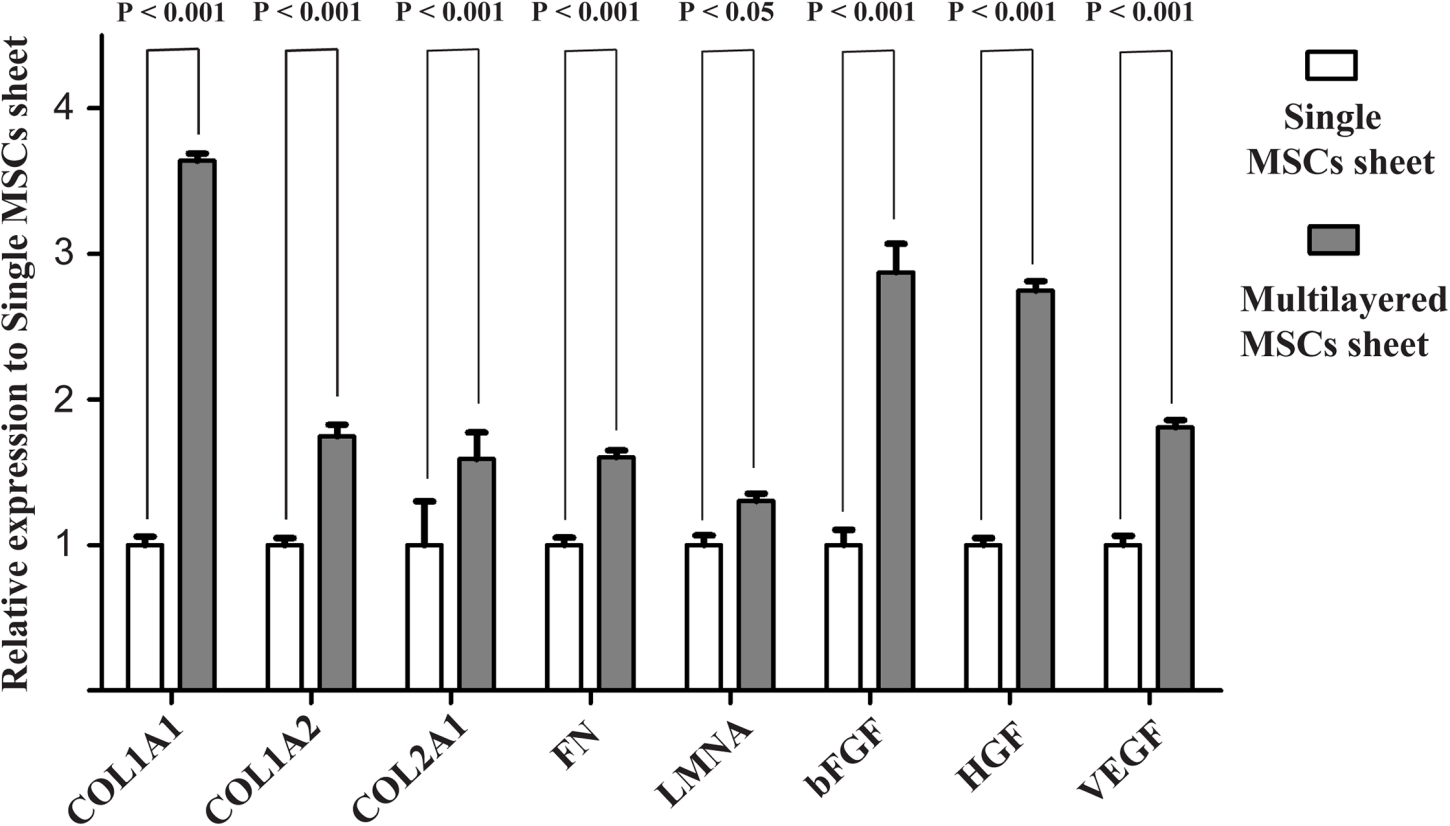
Real Time PCR analysis of *COL1A1*, *COL1A2*, *COL2A1*, *Fibronectin(FN)*, *LMNA*, *bFGF*, *HGF*, *VEGF* in the single and triple layers of MSCs cell sheet. Error bars indicate the s.d.; P, P-value.

**Fig 3:**
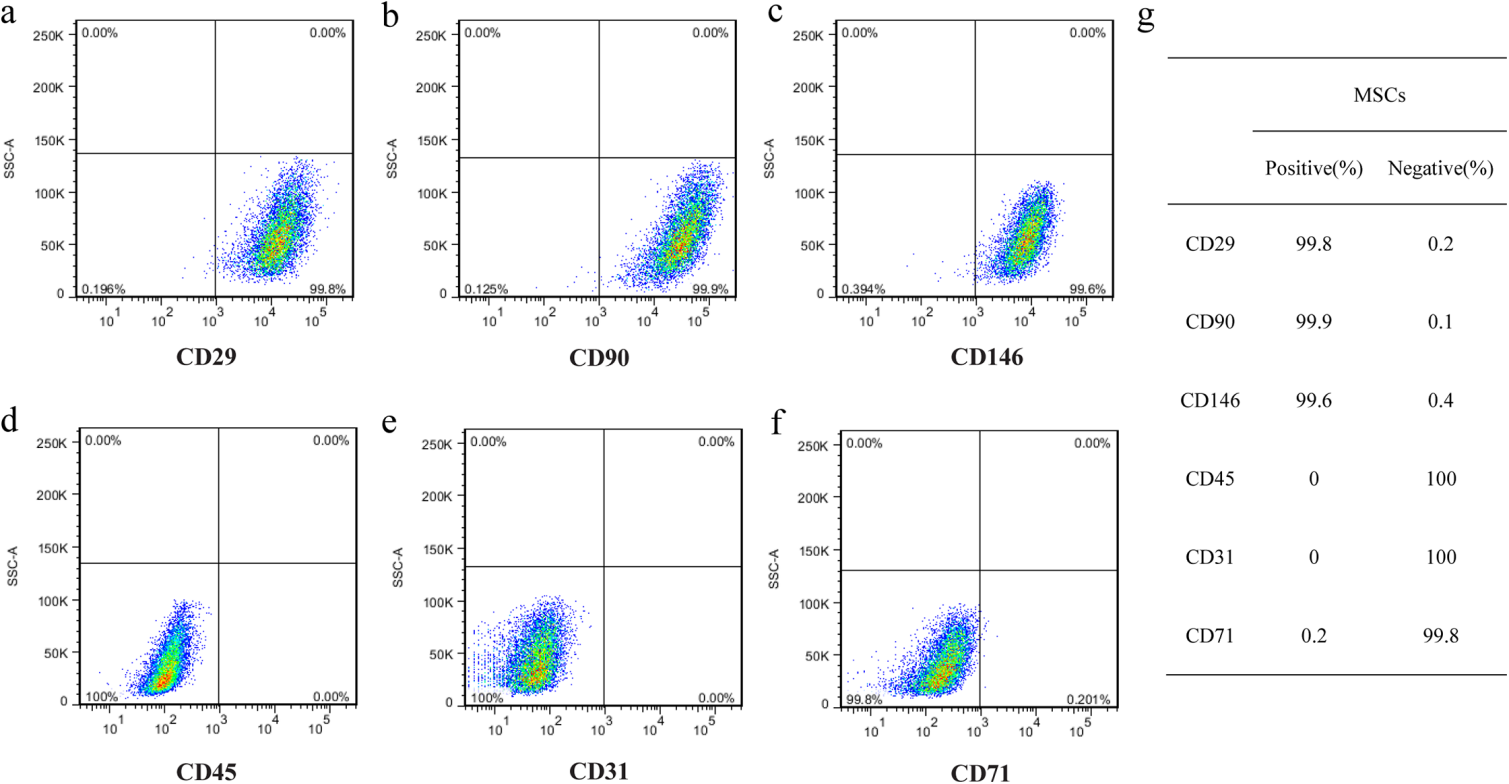
Flow cytometry analysis of the purity and pluripotency of MSCs. The surface marker of CD29(a), CD90(b), CD146(c), CD45(d), CD31(e), CD71(f) were analyzed and summarized(f).

Bone marrow-derived MSCs were cultured on temperature-responsive dishes at 37°C for 6–7 days for obtaining up to approximately 100% confluence. The confluent MSCs detached themselves as a monolayer cell sheet with intact cell-cell contacts (Fig 1A) after subsequent incubation at 20°C for 20–30 minutes. Triple-layered MSC sheet was obtained as an integrated tissue as shown in Fig 1B by the previously described stacking procedure. Each cell sheet was approximately 20 μm in diameter, and the thickness of the cell sheet was approximately 20 μm for a single layer (Fig 1C) and 60 μm for a triple layer (Fig 1D). Moreover, Real-time RT-PCR analysis demonstrated that some ECM and angiogenic factors were highly expressed in triple-layered MSC sheet compared with single cell sheet (Fig 2, S1 Table). MSCs were isolated from bone marrow on the basis of the adherent properties of these cells. After approximately three to four passages, most undifferentiated MSCs were positive for CD29 (Fig 3A), CD90 (Fig 3B) and CD146 (Fig 3C) markers, and the cells were also observed to be negative for CD45 (Fig 3D), CD31 (Fig 3E) a marker of vascular endothelial cells and CD71 (Fig 3F) by flow cytometric analysis (S1 Fig). Fig 3G shows summary of surface marker expression in MSCs. These results resembled those previously described for bone marrow–derived MSCs [13], indicating that the prepared MSCs sheet was composed of undifferentiated MSCs maintaining their multipotent characteristics. Thus, we confirmed that the majority of adherent cells isolated from bone marrow were MSCs.

### Direct adhesion of the Multilayered Cell Sheets to Native Heart Tissue

**Fig 4:**
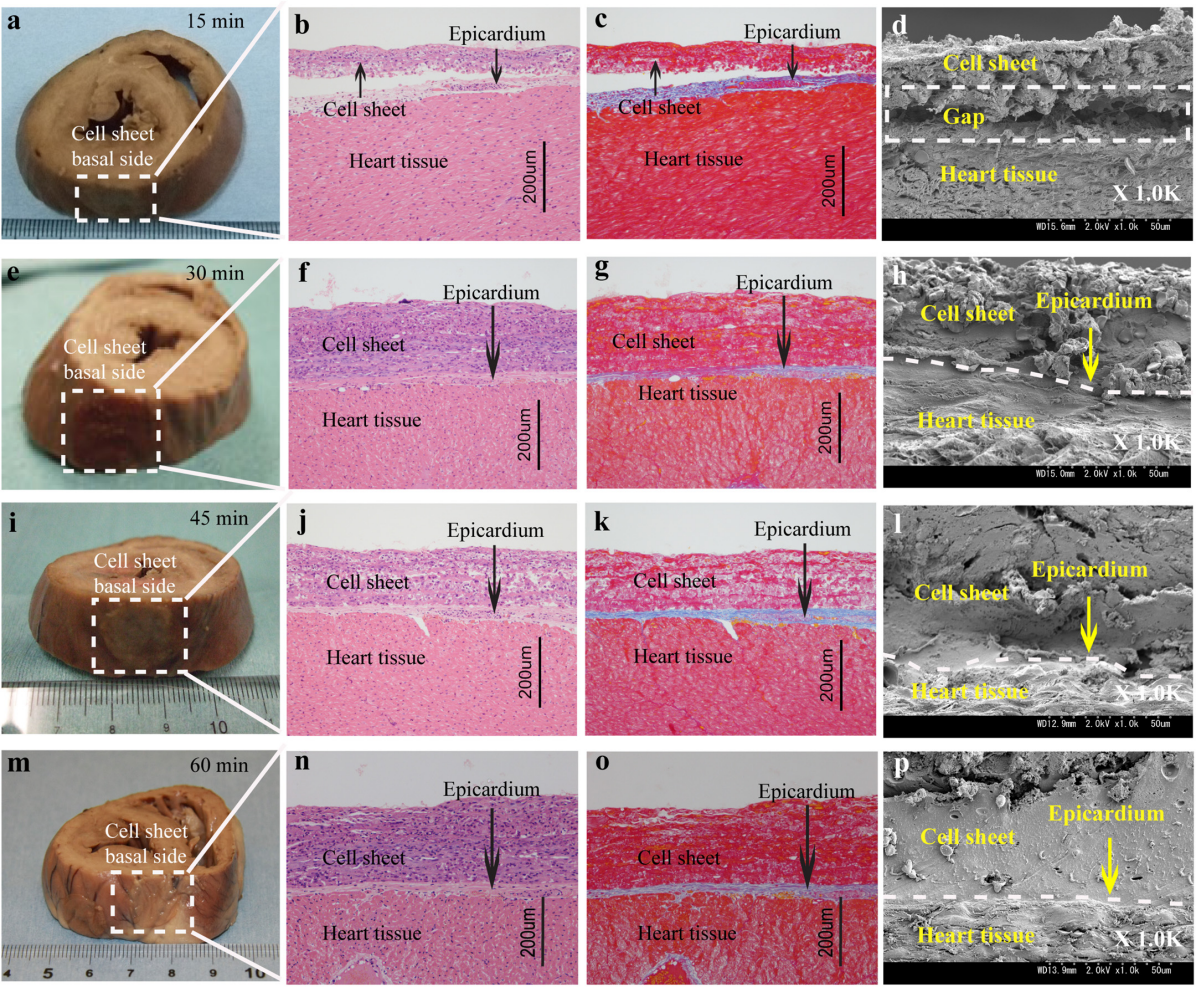
Direct basal side adhesion of MSCs sheet to the heart surface 15, 30, 45 and 30 minutes after transplantation. (a, e, i, m) Photographs of the heart tissue with cell sheet. The white squares show the physical contact of the transplanted cell sheet with the heart surface at 15 minutes (a), 30 minutes (e), 45 minutes (i) and 60 minutes (m). (b, c, f, g, j, k, n, o) Histological analysis of the adhesion part between the heart and the sheet by hematoxylin-eosin staining (b, f, j, n) and azan staining (c, j, k, o). Collagen-rich epicardium was identified as blue band by azan staining. (d, h, l, p) Images of scanning electron microscopy analysis of normal heart tissue after the transplantation of multilayered cell sheets with the basal side with a magnification of 1000-fold. The area marked with the white dot-line empty is a gap space between the cell sheet and the heart tissue. Scale bars in b, c, f, g, j, k, n, and o are 200 μm.

To identify the transplanted multilayered MSCs cell sheet onto heart tissue, a triple-layered MSCs cell sheet was transplanted to exposed porcine heart with the basal side attached to the surface of heart tissue 15 minutes to 60 minutes (n = 5) time periods. Although the cell sheets transplanted for the time periods maintained physical contact with the heart tissue (Fig 4A, 4E, 4I and 4M), histological and scanning electron microscope analyses showed distinct differences for each time period. At 15 minutes after the cell sheet transplantation, only a minor part of the cell sheets was in contact with the epicardium of the heart, and there was an extensive gap that existed between the transplanted cell sheets and the epicardium of heart (Fig 4B, 4C and 4D). These results indicated that the transplanted cell sheets failed to efficiently attachment with the heart tissue within 15 minutes. However, at 30 minutes after transplantation, the adhesion between the cell sheets and the heart tissue was clearly observed (Fig 4F, 4G and 4H). Moreover, the adhesion was also observed after the increase of incubation time from 30 to 45 or to 60 minutes after cell sheet transplantation (Fig 4J, 4K and 4L) (Fig 4N, 4O and 4P), and it had no effect on the final tissue morphology around the adhesion region. Therefore, the minimum time for adequate adhesion was judged to be 30 minutes after cell sheet transplantation in a large animal model. This time period is roughly congruent to the time period for closing the chest during open heart surgery.

### Inverse attachment of the multilayered cell sheets to native heart tissue

**Fig 5:**
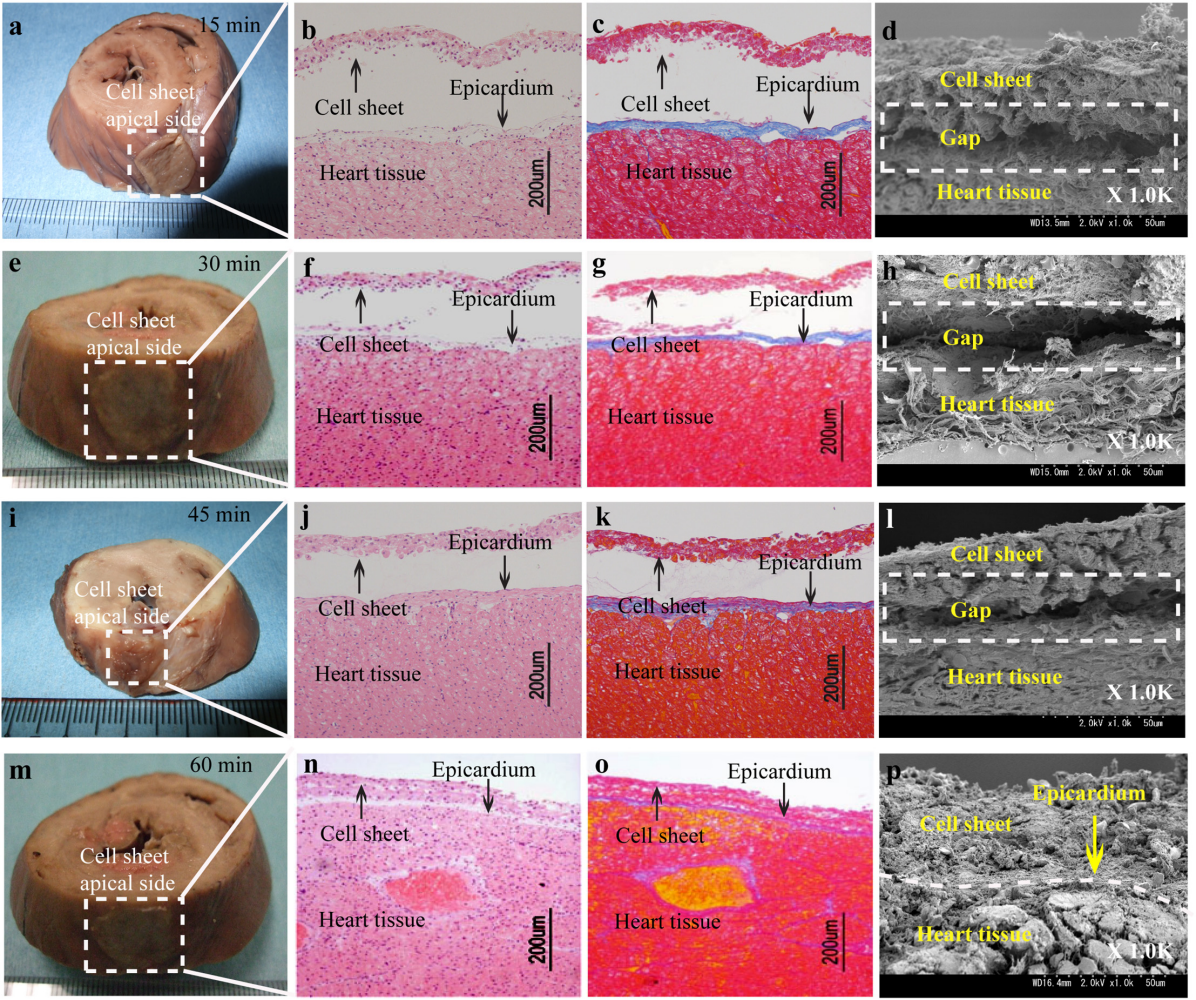
Inverse attachment of MSCs sheet onto the heart surface 30 and 60 minutes after transplantation. (a, e, i, m) Photograph of the heart tissue. The white squares show the physical contact of transplanted the cell sheet with the heart surface. (b, c, f, g, j, k, n, o) Histological analysis of the adhesive part between the heart tissue and the cell sheet by hematoxylin-eosin staining and azan staining. Collagen-rich epicardium was identified as blue by azan staining. (d, h, l, p) Images of scanning electron microscopy analysis of normal heart tissue after the transplantation of multilayered cell sheets with the apical side attached to the heart tissue with a magnification of 1000-fold. The area marked with the white dot-line is a gap space between the cell sheet and the heart tissue. Scale bars in b, c, f, g, j, k, n, and o are 200 μm.

**Fig 6:**
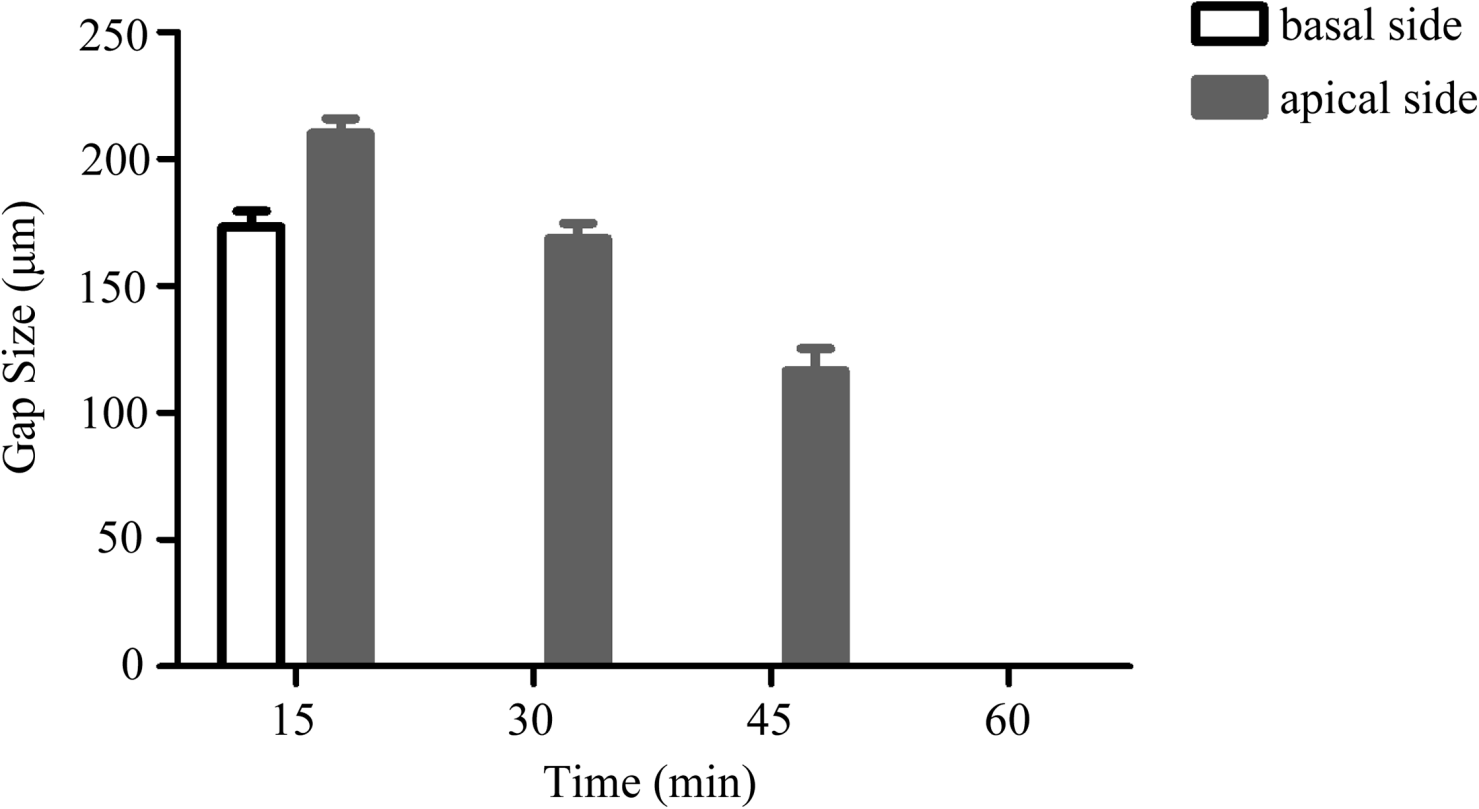
The measurement of gap size between the transplanted cell sheets(basal side and apical side) and the epicardium at 15 minutes, 30 minutes, 45 minutes and 60 minutes. The gap size were measured and confirmed by three analysis of hematoxylin-eosin staining. Error bars indicate the s.d.

To obtain and determine the most optimal full adhesion between cell sheet and the host heart surface, the application of cell-sheet surface consisting of both apical and basal surfaces had to be evaluated. To determine which side of cell-sheet surface application was best, the effect of inverse attachment strategy was performed. When multilayered cell sheets were transplanted to porcine heart with the apical side attached to the heart tissue, our investigation confirmed that the application of the basal side of cell sheets was crucial for successful transplantation (n = 5). The inverse attachment strategy, or application of the apical side, required longer time for having the optimum attachment of the cell sheets to the heart tissue when compared with the direct basal side adhesion strategy. At 15, 30 and 45 minutes after cell sheet transplantation, although the cell sheets were found to adhere physically with apical side application (Fig 5A, 5E and 5I), histological and scanning electron microscope analyses indicated that the transplanted cell sheet remained almost totally separated from the heart tissue after cell sheet transplantation (Fig 5B, 5C and 5D) (Fig 5F, 5G and 5H) (Fig 5J, 5K and 5L). Naturally, a prolonged time period of 60 minutes allowed the sheets to adhere without an obvious gap between the transplanted cell sheets and the heart tissue. The cell sheets were observed to adhere firmly not only by the eye (Fig 5M), but also by histological and scanning electron microscope analyses (Fig 5N, 5O and 5P). Moreover, the size of the gaps at the epicardial surface has been measured to objectively show that basal side of cell sheet was better than apical side (Fig 6, S2 Table). These agree with the established fact that the basal side of the MSCs multilayered sheet significantly enhanced the adhesion of the cell sheet to the heart tissue compared with the apical side in a shorter amount of time.

## Discussion

Cell therapy has been progressing rapidly using the direct injection in clinical studies for the treatment of heart failure. However, it may be limited by failure to regenerate cardiac tissue [4]. Since application of cell sheets allows for cell-to-cell connections and can be completely transplanted into the damaged heart without losing cells, cell sheet technology is described as a suitable strategy for tissue repair and regenerative medicine in research and appropriate clinical studies [28–31]. The cell sheet technique also has been further developed along with several tissue-engineering applications such as fabrication of pulsatile cardiac tissue grafts, myocardial tissue reconstruction by using temperature-responsive culture dishes [6, 13, 14, 32–35]. The objective of the study was to investigate the adhesion time course of transplanted cell sheet on heart tissue in order to find the time requirement for adequate adhesion. This information is critical for successful clinical application in open-heart surgery using this technology.

Recently, studies have shown that multilayered cell sheets prepared from multipotent MSCs may be a useful technology for tissue regeneration, considering that nearly all organs are complicated multilayer tissues composed of various cell types such as adipose tissue-derived mesenchymal stem cells [11], marrow stoma cells [31], et al. In this study, the successful culture of multilayered MSCs sheet and their adhesion to native porcine heart tissue represented an important step in the clinical application of cell sheet technique to cardiac surgery and cardiovascular repair. Additionally, we have previously reported that mesenchymal stem cell sheets can be used to facilitate the repair of the scarred myocardium after myocardial infarction (MI) in rats [11]. This technique was applied to porcine heart as a large animal model, which is regarded as more similar to the human heart, for obtaining further confirmation of the clinical feasibility of this technique [36].

Most importantly, operating on the living heart has inherent risks, and opening the chest cavity leads to the exposure of the cardiovascular organs increasing the risks of heart failure. In addition, the risk of infection and other adverse effects are also increased by prolonging open-heart surgery. The duration of open-heart surgery affects the post-operative recovery of patients. For this reason, it is necessary to minimize operation time as short as possible after cell sheet transplantation. On the other hand, it is important to ensure the adequate adhesion of the cell sheets onto the heart tissue before the chest is closed. If the cell sheet adheres onto the heart tissue incompletely, closing the chest too early might impair the survival and growth of the implant and lead to unsuccessful cell sheet transplantation. This study confirmed that a specific time period of 30 minutes was required for cell-sheet basal side adhesion to the heart tissue after cell sheet transplantation in pig models. This allows cardiac surgeons to know the optimal time to close the chest after cell sheet transplantation. This research also provided a guide for further work in the area of MSCs sheet-based treatment for damaged heart tissue in clinical studies.

Moreover, when a triple-layered MSCs sheet was transplanted to the heart tissue with the basal side and apical side separate for 15 minutes, histological and scanning electron microscope analyses showed space between the cell sheet and the heart tissue (Fig 4B–4D), indicating proteins in ECM such as collagen, fibronectin, laminin, vitronection, small matricellular proteins, et al. had still been unable to bind completely to the heart tissue. However, 30 minutes after cell sheet basal side transplantation, cell-adhesion related proteins in ECM on the cell sheet were speculated toward the interface [37] and the cell sheet completely adhered to the heart tissue as demonstrated by physical adhesion (Fig 4E) and by biological connection (Fig 4F–4H). Moreover, 45 minutes and 60 minutes after cell sheet transplantation, the cell sheet still completely adhered to the heart tissue (Fig 4I–4P). It was shown that 60 minutes after cell sheet transplantation, the apical cell sheet adhered to the heart tissue adequately but this time period is inappropriate in an open heart surgical setting. (Fig 5M–5P). We speculated ECM moved from the basal side to the apical side with additional time allowing adhesion to the surface of the heart tissue after the cell sheet transplantation. This study also indicates that the existence of ECM proteins facilitated the attachment process between cell sheet and the heart tissue in a shorter amount of time.

Our results also indicated that a longer time was necessary for generating a stronger adhesion between the cell sheet and the heart tissue. However, the prolongation of operation time for open-heart surgery is generally known to lead to higher post-surgical complications. Therefore, it is important to determine the required time for generating the stronger adhesion between the cell sheets and the heart tissue necessary to ensure safer open-heart surgery. ECM proteins increased the adhesion strength and reduced the minimum required time for the attachment of cell sheet to the heart tissue [38–40]. We may be able to use a membrane to facilitate transplantation by placing the membrane on the top of the cell sheet for collection, and then transplantation to the heart surface. These effects should be investigated in further studies.

In summary, our study identified the minimal time for the adhesion of basal side of multilayered MSCs cell sheet to the heart tissue was 30 minutes. Also important, the results of our present study provide information to better guide the time period for closing the chest after cell sheet transplantation during an open heart surgery.

## Conclusions

This preliminary study shows clear progress toward the clinical application of MSC multilayered cell sheets as a potential treatment vehicle for human heart disease. The great promise of cell sheet technology is expected to further benefit human health in the future.

## Supporting Information

S1 Fig The detail of flow cytometry analysis of the purity and pluripotency of MSCs. The surface marker of CD29, CD90, CD146, CD45, CD31, CD71 were analyzed.
(TIF)

S1 Table The relative expression value of genes for singe and multilayered MSCs sheet in the Real Time PCR. (DOCX)

S2 Table The value of gap size between the transplanted cell sheets (basal side and apical side) and the epicardium at 15, 30, 45 and 60 minutes. (DOCX)
