# Supplementary figures and images for: Time Course of Cell Sheet Adhesion to Porcine Heart Tissue after Transplantation
Source: PLoS One. 2015 Oct 7;10(10):e0137494. doi: 10.1371/journal.pone.0137494 (PMC4596823; doi:10.1371/journal.pone.0137494)

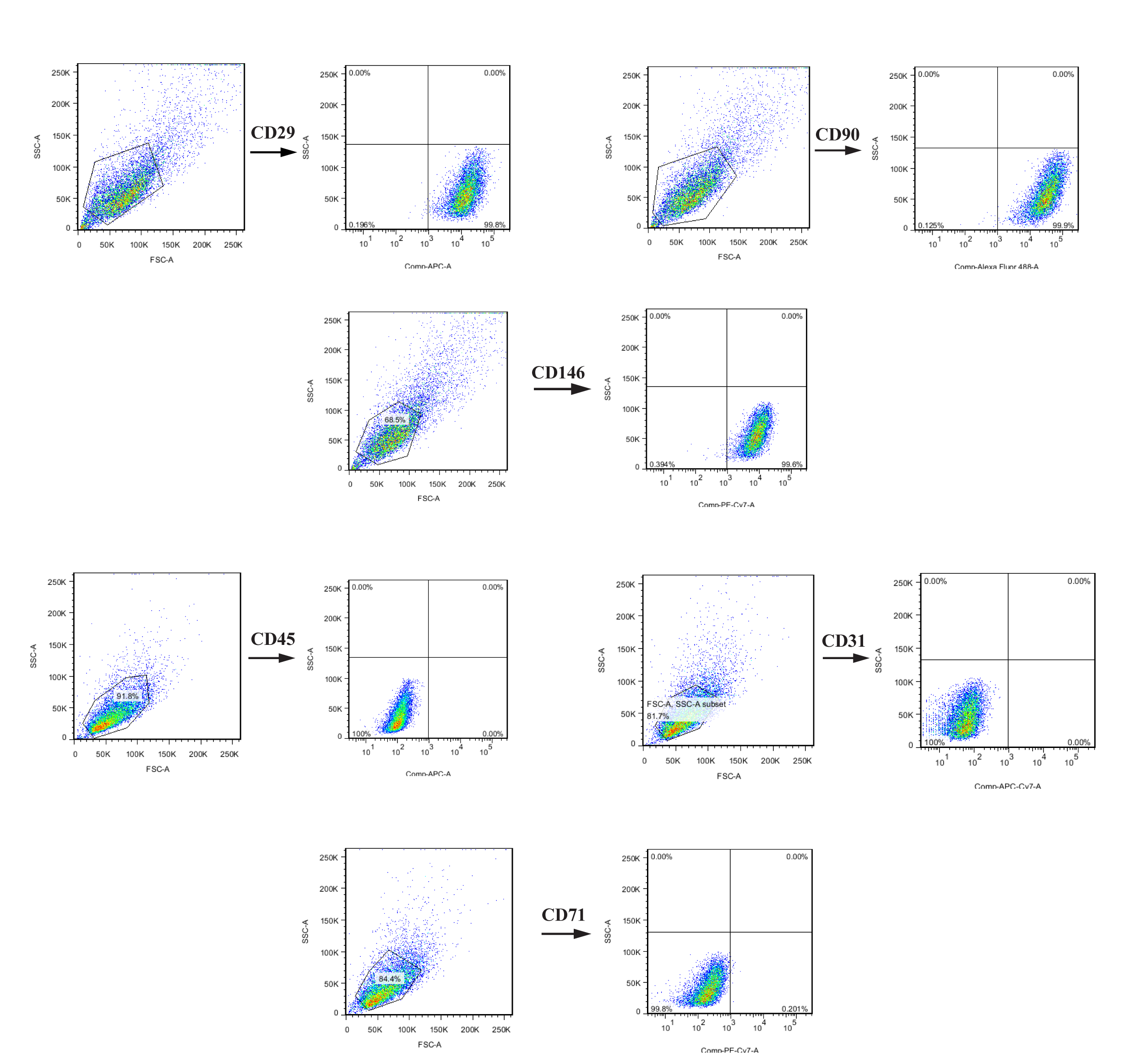

Supplement: S1 Fig — The surface marker of CD29, CD90, CD146, CD45, CD31, CD71 were analyzed. (TIF) [file pone.0137494.s001.tif]
