# Supplementary material for: Time Course of Cell Sheet Adhesion to Porcine Heart Tissue after Transplantation
Source: PLoS One. 2015 Oct 7;10(10):e0137494. doi: 10.1371/journal.pone.0137494 (PMC4596823; doi:10.1371/journal.pone.0137494)
Supplement: S1 Table — (DOCX) [file pone.0137494.s002.docx]

**S1 Table: The relative expression value of genes for singe and multilayered MSCs sheet in the Real Time PCR .**

| Gene | Single MSCs sheet | | Multilayered MSCs,sheet | |
| --- | --- | --- | --- | --- |
|  | RQ(Relative Quantification) | S.D. | RQ(Relative Quantification) | S.D. |
| *COL1A1* | 1 | 0.057 | 3.64 | 0.05 |
| *COL1A2* | 1 | 0.049 | 1.75 | 0.08 |
| *COL2A1* | 1 | 0.30 | 1.59 | 0.18 |
| *FN1* | 1 | 0.05 | 1.60 | 0.05 |
| *LMNA* | 1 | 0.06 | 1.30 | 0.05 |
| *bFGF* | 1 | 0.10 | 2.87 | 0.20 |
| *HGF* | 1 | 0.05 | 2.75 | 0.06 |
| *VEGF* | 1 | 0.06 | 1.81 | 0.05 |
