# Supplementary material for: Time Course of Cell Sheet Adhesion to Porcine Heart Tissue after Transplantation
Source: PLoS One. 2015 Oct 7;10(10):e0137494. doi: 10.1371/journal.pone.0137494 (PMC4596823; doi:10.1371/journal.pone.0137494)
Supplement: S2 Table — (DOCX) [file pone.0137494.s003.docx]

**S2 Table: The value of gap size between the transplanted cell sheets(basal side and apical side) and the epicardium at 15min, 30min, 45min and 60min.**

|  | 15min | Average | S.D. | 30min | Average | S.D | 45min | Average | S.D | 60min | Average | S.D |
| --- | --- | --- | --- | --- | --- | --- | --- | --- | --- | --- | --- | --- |
| Basal gap size(μm) | 180 | 173.33 | 6.11 | 0 | 0 | 0 | 0 | 0 | 0 | 0 | 0 | 0 |
|  | 172 |  |  | 0 |  |  | 0 |  |  | 0 |  |  |
|  | 168 |  |  | 0 |  |  | 0 |  |  | 0 |  |  |
| Apical gap size(μm) | 210 | 210 | 6 | 175 | 168.67 | 6.03 | 115 | 116.67 | 8.62 | 0 | 0 | 0 |
|  | 204 |  |  | 168 |  |  | 109 |  |  | 0 |  |  |
|  | 216 |  |  | 163 |  |  | 126 |  |  | 0 |  |  |
